# Modeling and measuring intracellular fluxes of secreted recombinant protein in *Pichia pastoris *with a novel ^34^S labeling procedure

**DOI:** 10.1186/1475-2859-10-47

**Published:** 2011-06-26

**Authors:** Martin Pfeffer, Michael Maurer, Gunda Köllensperger, Stephan Hann, Alexandra B Graf, Diethard Mattanovich

**Affiliations:** 1 University of Natural Resources and Life Sciences, Department of Biotechnology, Muthgasse 18, 1190 Vienna, Austria; 2 University of Applied Sciences FH-Campus Vienna, School of Bioengineering, Muthgasse 18, 1190 Vienna, Austria; 3 University of Natural Resources and Life Sciences, Department of Chemistry, Muthgasse 18, 1190 Vienna, Austria; 4 Austrian Centre of Industrial Biotechnology (ACIB GmbH), Muthgasse 11, 1190 Vienna, Austria

## Abstract

**Background:**

The budding yeast *Pichia pastoris *is widely used for protein production. To determine the best suitable strategy for strain improvement, especially for high secretion, quantitative data of intracellular fluxes of recombinant protein are very important. Especially the balance between intracellular protein formation, degradation and secretion defines the major bottleneck of the production system. Because these parameters are different for unlimited growth (shake flask) and carbon-limited growth (bioreactor) conditions, they should be determined under "production like" conditions. Thus labeling procedures must be compatible with minimal production media and the usage of bioreactors. The inorganic and non-radioactive ^34^S labeled sodium sulfate meets both demands.

**Results:**

We used a novel labeling method with the stable sulfur isotope ^34^S, administered as sodium sulfate, which is performed during chemostat culivations. The intra- and extracellular sulfur 32 to 34 ratios of purified recombinant protein, the antibody fragment Fab3H6, are measured by HPLC-ICP-MS. The kinetic model described here is necessary to calculate the kinetic parameters from sulfur ratios of consecutive samples as well as for sensitivity analysis. From the total amount of protein produced intracellularly (143.1 μg g^-1 ^h^-1 ^protein per yeast dry mass and time) about 58% are degraded within the cell, 35% are secreted to the exterior and 7% are inherited to the daughter cells.

**Conclusions:**

A novel ^34^S labeling procedure that enables *in vivo *quantification of intracellular fluxes of recombinant protein under "production like" conditions is described. Subsequent sensitivity analysis of the fluxes by using MATLAB, indicate the most promising approaches for strain improvement towards increased secretion.

## Background

The production of recombinant proteins in yeast has to compete with other host organisms, mainly bacteria and mammalian cell lines. Strain improvement therefore is an essential step between the discovery of a new protein and its large scale production. Yeasts like *Pichia pastoris *grow faster and to a higher cell density compared to mammalian cells, however the low specific productivity (the amount of secreted protein per unit biomass and time) is their major drawback [1]. A lot of efforts have already been made to find and overcome specific bottlenecks in the cellular protein production and secretory system [reviewed by [2]].

At genomic level increasing the gene copy number as well as the promoter strength leads to higher productivities [3-5]. The overload of the endoplasmic reticulum (ER) with recombinant protein may induce the unfolded protein response (UPR) [6-8] followed by enhanced ER-associated degradation (ERAD) [9,10]. Among many other things, UPR reduces overall translation speed [11] and enforces ERAD via the Ire1 signaling cascade [12]. ERAD causes proteolytic digestion of malfolded protein in the cytosolic proteasome [13]. Thus, reduced ER-stress can be beneficial for recombinant protein production. Therefore, many attempts have been made to improve the complex process of protein maturation, mainly by co-overexpressing ER resident chaperons or foldases like BiP / Kar2, Pdi1 or calnexin [14-16]. Furthermore the transport from the ER to the Golgi and finally into the exterior can be improved by co-overexpression of proteins involved in this pathways. Examples are Sso1 and Sso2, both coding for plasma membrane t-SNARE proteins [17] or Cog6, Coy1 and Bmh2, all coding for proteins involved in vesicular transport [18].

In the strain improvement process by cell engineering it is required to achieve high yields in short time. A focused and systematic approach therefore would be to identify the most important bottleneck in recombinant protein synthesis being the one which modification has the highest impact on protein titers.

Kinetic models are a valuable tool in this regard, as they give insights into intracellular fluxes. The formal kinetic description of the processing and transport of secreted proteins are already known for quite a while [19,20]. However, the challenge is the experimental determination of the parameters needed in those models. Furthermore it is necessary to make as few assumptions as possible so that a production process can still be described. In this regard the experiments have to be done under carbon limited, production "similar", growth in bioreactors under defined and controlled conditions instead of using shake flask cultivations. This is usually not possible when labeling is performed with radioactive isotopes or when protein kinetics is measured with microscopic tools, like fluorescence microscope imaging. Handling of large volumes of radioactive material is not feasible for risk of contamination. Microscopic imaging on the other side quantifies the protein fluxes by comparing images of living cells over time [21]. The advantage is that single cells are analyzed instead of an average. However cells are exposed to non-defined conditions which are likely to be different to the bioreactor. Furthermore, this method is limited to fluorescent or fluorescent-tagged proteins.

It is important that the model of choice accounts for intracellular degradation as well, because a substantial amount of recombinant protein may be degraded via ERAD or other pathways. Also the dilution by growth, especially in the fast growing prokaryotes and yeast cells, has to be taken into account. For example at a specific growth rate μ = 0.1 h^-1^, 7% of the intracellular protein is inherited to the daughter cells (as described in this study). Several kinetic model studies for antibody production have been developed [22-25], but intracellular protein degradation as well as protein inheritance to the daughter cells was not taken into account in these cases.

In this work we present a novel ^34^S labeling method during chemostat cultivation, providing data to consider intracellular protein formation, intracellular degradation, secretion and dilution by growth in the kinetic model. Further sensitivity analysis of the intracellular protein flux enables the estimation of their impact and serves as a decisions basis for further strain improvement.

## Results

### Structured Kinetic Model

The structured kinetic model describes the intra- and extracellular recombinant protein pools. The dynamics of the intra- and extracellular Fab3H6 pools are expressed in two separate differential equations (1) and (3), which are modified from Noe and Delenick [19] and Batt and Kompala [20].

The amount of intracellular protein (Pi) depends on the fluxes of intracellular protein formation (q_Pi_), protein secretion (q_Sec_), intracellular protein degradation (q_Deg_) and protein dilution into the daughter cells (q_Dil_). Due to the rapid growth of yeast cells, the inheritance of protein to daughter cells has to be taken into account. The dynamics of protein secretion, degradation and dilution can be assumed to be a first-order kinetic [22,23,26], with specific time constants (K). Equation (1) can therefore be transformed into equation (2).(1)(2)

In chemostat culture, the extracellular product pool (Pe) is a function of the secreted protein (q_Sec_) and the amount of protein in the media that is harvested (q_Har_) (equation 3). Also here first-order kinetic can be assumed and equation (3) transformed into equation (4).(3)(4)

The observed viability of more than 99.0% ensures that the dilution rate (D) and the specific growth rate (μ) are equal in these continuous cultivations and thus also the time constants of dilution K_Dil _and harvest K_Har _are the same.

### Calculation of the time constants for secretion K_Sec _and degradation K_Deg_

Due to steady state conditions during continuous cultivation no change in the total amount of P_i _or P_e _occurred over time. Therefore equation (4) is zero and can be transformed into equation (5).(5)

To determine the time constant for protein degradation K_Deg_, the decrease of the amount of ^32^S-containing protein (P^32^S) during the continuous labeling experiment with ^34^S is used. The fraction of non-labeled sodium sulfate during labeling remains always below 1%. It is therefore assumed that the intracellular ^32^S protein formation during labeling is negligible. Thus equation (2) can be converted to equation (6) and further simplified for q_(Pi32S) _(equation 7). The solution of this new differential equation can be transformed into a linear system, shown in equation (8).(6)(7)(8)

Linear regression analysis was performed with the statistics software package R. The slope represents the sum of the three time constants for degradation, secretion and dilution. K_Deg _is calculated by subtracting the known K_Sec _and K_Dil _from the absolute value of the slope.

### Calculation of the rate of intracellular recombinant protein formation (q_Pi_)

Due to steady state considerations, equation (1) can be set to zero. The result is shown in equation (9), were q_Pi _is depicted. This equation expresses that all protein that is secreted, degraded or transferred into daughter cells is produced in the cells before.(9)

### Chemostat cultivation

Extracellular and intracellular Fab3H6 concentrations remained constant over time (data not shown). The average concentrations were 91.8 μg per gram YDM (STD: 9.6%) intracellular and 507.5 μg per gram YDM extracellular (STD: 12.1%). The standard deviation (STD) derived from two independent chemostat cultivations.

### Immunoprecipitation

**Figure 1:**
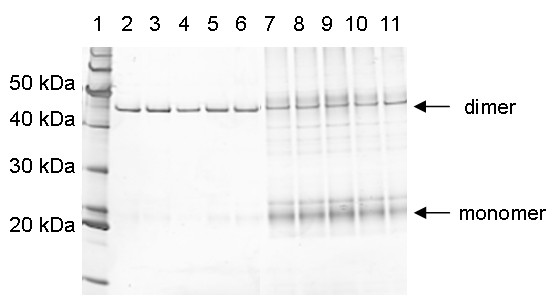
**Immunoprecipitation of Fab3H6**. Immunoprecipitation of extracellular and intracellular Fab3H6 during ^34^S-labeling. Lanes 2-6: consecutive extracellular samples, lanes 7-11: consecutive intracellular samples. Immunoprecipitates were separated by non-reduced SDS-PAGE and silver stained. Dimers and monomers of the Fab fragment are indicated by arrows. The protein ladder (Page Ruler, Invitrogen) is seen in lane 1.

The antibody fragment Fab3H6 was immunopurified during the continuous labeling from the fermentation supernatants and the cell biomass respectively (figure 1). In the supernatant almost only the dimer was present, whereas the cell lysate contained a substantial amount of monomers. Some impurities were detected in the immunoprecipitates (IPs) from the cell lysates. This might be a result of covalent and hydrophobic interactions of the nascent peptide chains during folding, assembly and transport. Therefore the decrease of the intracellular ^32^S / ^34^S ratio has to be corrected by subtracting the background levels derived from impurities. According to the observed half times of secretion and degradation (table 1), all intracellular recombinant protein should contain less than 0.1% ^32^S 140 min post labeling. Therefore it is assumed that from this time point on the measured ^32^S signal derived from impurities. The background was calculated by extrapolating the ^32^S / ^34^S ratios from 140 min post labeling on towards the beginning of the labeling.

**Table 1:** Intracellular fluxes of the antibody fragment Fab3H6

*flux*	*rate (q)*	*time constant (K)*	*half time (t_1/2_)*
intracellular protein formation (Pi)	143.1 μg g^-1 ^h^-1 ^(STD 8.8%)		
intracellular protein degradation (Deg)	83.3 μg g^-1 ^h^-1 ^(STD 9.3%)	0.0151 min^-1 ^(STD 2.8%)	45.8 min (STD 2.8%)
protein secretion (Sec)	50.7 μg g^-1 ^h^-1 ^(STD 12.1%)	0.00920 min^-1 ^(STD 2.5%)	75.3 min (STD 2.5%)
dilution by growth (Dil)	9.18 μg g^-1 ^h^-1 ^(STD 9.6%)	0.00167 min^-1 ^(STD 0.05%)	415.9 min (STD 0.05%)

The intracellular fluxes are represented as rates q (μg Fab3H6 (g YDM)^-1 ^h^-1^), time constants K (min^-1^) or half times (min). STD = standard deviation; n = 2 independent replicate chemostat cultivations.

### Sulfur isotopic distribution during labeling

**Figure 2:**
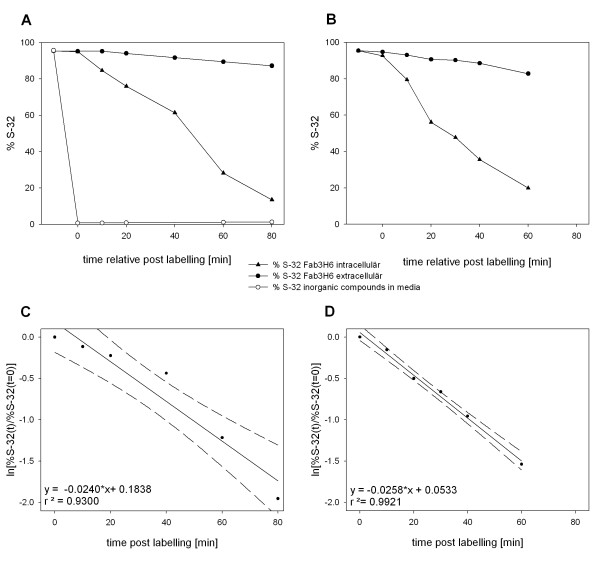
**^34^S labeling in chemostat culture**. (A + C) and (B + D) respectively derive from two independent, replicate fermentations. In (A) and (B) the decrease of the ^32^S content of intra- and extracellular Fab3H6 during continuous ^34^S labeling in chemostat cultivation is shown. Additionally, in (A) the ^32^S content of small compounds, mainly sulfate, in the media was measured (open circles). The linear regression analysis of the normalized ^32^S contents of the intracellular Fab3H6 (equation 8) is presented in (C) and (D). The dashed lines illustrate the 95% confidence intervals. The slope represents the sum of the time constants of protein secretion, degradation and dilution by growth.

The ^32^S amount of compounds smaller than 3 kDa, mainly sulfate, in the media is shown in figure 2A (open circles). The values are always around or below 1%, which is a prerequisite for the kinetic model (equation 7). The decrease of the unlabeled antibody fragment Fab3H6 from two replicative, independent chemostat cultivations is shown in figure 2A and 2B. The amount of intracellular non-labeled ^32^S recombinant protein is always below the extracellular value. This is not surprising because labeled protein has to be produced within the cell before being released to the media. In some samples the signal to noise ratio was not sufficient for ICP-MS measurement so that these were not taken into account. Therefore, the number of data points in figure 2A and figure 2B are not identical.

### Intracellular Fab3H6 fluxes

The dilution rate of the chemostat defines the growth rate of the culture. The specific growth rate expressed in minutes equates the time constant of the dilution by growth K_Dil _= 0.00167 min^-1 ^(table 1). The corresponding half time t_1/2 Dil _= 415.9 min is calculated by equation (10):(10)

The relative standard deviation (STD) of 0.05% results from the discontinuous harvest (see material an methods). The rate by which the protein is inherited to the daughter cells q_Dil _= 9.18 μg g^-1 ^h^-1 ^(STD: 9.6%).

For the determination of K_Sec _the quotient from extra- and intracellular protein concentration is multiplied with K_Dil _(equation 5). K_Sec _was calculated to be 0.0092 min^-1 ^(STD: 2.5%) with the corresponding half time t_1/2 Sec _= 75.3 minutes (STD: 2.5%) and the secretion rate q_Sec _= 50.7 μg g^-1 ^h^-1 ^(STD: 12.1%) (table 1).

Before the degradation rate can be calculated, the intracellular values of the unlabeled ^32^S Fab3H6 content (see figure 2B) have to be transformed according to equation (8). This is done by taking the logarithm of the normalized data points. The linear regression analyses of the two biological replicates are shown in figure 2C and figure 2D respectively, with the transformed values on the y-axis and the time on the x-axis. K_Deg _is calculated by subtracting K_Sec _and K_Dil _from the positive slopes 0.0240 min^-1 ^and 0.0258 min^-1^. The time constant for intracellular degradation K_Deg _= 0.0151 min^-1 ^(STD 2.8%) with the corresponding t_1/2 Deg _= 45.8 min (STD 2.8%) and q_Deg _= 83.3 μg g^-1 ^h^-1 ^(STD: 9.3%). Furthermore, the 95% confidence interval is represented by the dashed line in figure 2D.

The rate of intracellular protein formation q_Pi _is simply the sum of q_Dil_, q_Sec _and q_Deg _and is 143.1 μg g^-1 ^h^-1 ^(STD: 8.8%).

**Figure 3:**
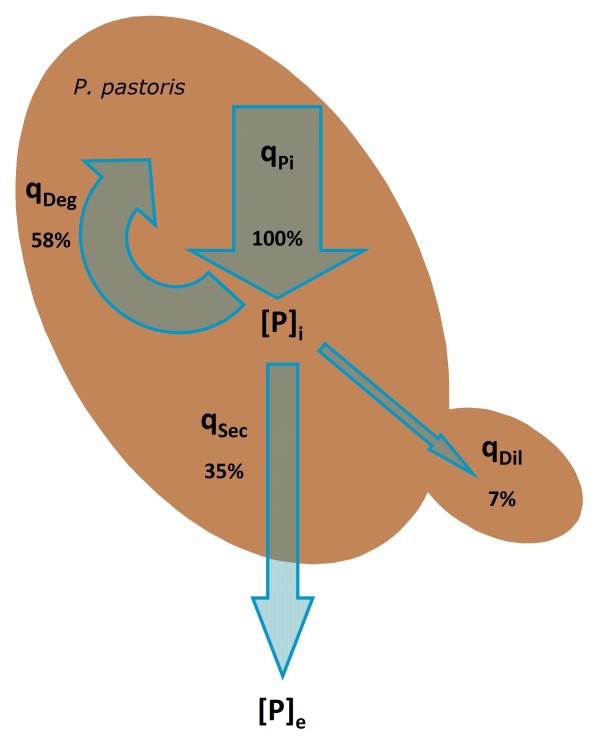
**Overview of the intracellular fluxes of the recombinant antibody fragment Fab3H6 in *P. pastoris***.

Figure 3 gives an overview of the rates, where q_Pi _of 143.1 μg g^-1 ^h^-1 ^represent 100%. The other rates (q_Deg_, q_Sec _and q_Dil_) are shown relative to the intracellular protein formation. From 100% being produced within the cell, 58% are degraded intracellularly, 35% are secreted to the exterior and 7% are inherited to daughter cells.

### Sensitivity analysis

**Figure 4:**
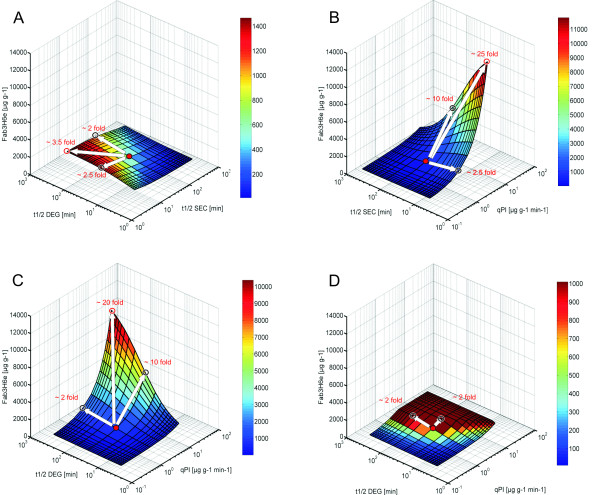
**Sensitivity analysis of the extracellular Fab3H6 concentration**. In each picture the effect of two different input variables on the extracellular Fab3H6 concentration is shown. The red dot in the middle represents the measured values in the chemostat. These initial values were changed from -10 to +10 fold resulting in depicted areas. In (A) t_1/2 Deg _and t_1/2 Sec_, in (B) t_1/2 Sec _and q_Pi_, in (C) and (D) t_1/2 Deg _and q_Pi _were analyzed. Furthermore, in (D) the maximum secretion capacity was set to 101.4 μg g^-1 ^h^-1 ^(2 fold of the measured value).

The sensitivity analysis reflecting the impact of the different model parameters to the extracellular Fab3H6 concentration is shown in figure 4. Parameters, describing either protein formation (q_Pi_), secretion (t_1/2 Sec_) or degradation (t_1/2 Deg_) are continuously changed in MATLAB from their starting point up to +/- 10 fold. Each fold increase or decrease is pictured as a solid line. In the three dimensional plots, two of the model parameters are plotted against the corresponding Fab3H6 titers, resulting in the presented areas (figure 4A-D). The 10 fold improvement of each parameter is shown as a white arrow.

A 10 fold "faster" secretion, t_1/2 Sec _= 7.5 min, would increase extracellular Fab3H6 concentration by the factor 2.5. A 10 fold "improved" degradation, t_1/2 Deg _= 458 min, results in a 2.0 fold increase. By combining both effects the titers are estimated to be 3.5 fold higher (figure 4A).

The intracellular protein formation rate q_pi _has an almost linear effect on the extracellular protein concentration (figure 4B and figure 4C). By increasing q_Pi _by the factor of 10, from 143.1 μg g^-1 ^h^-1 ^to 1.43 mg g^-1 ^h^-1^, the predicted Fab3H6 concentrations are also enhanced by approximately the same factor to about 5000 μg g^-1^.

In figure 4A-C no limit in the secretion pathway was anticipated in the underling kinetic model. This assumption however is not realistic, especially in yeast cells. It has been reported many times that the secretory capacity can be a bottleneck during recombinant protein production [2,4,14,18]. Therefore, an additional model constraint was implemented in figure 4D, where the maximum secretion rate was set to be twice the default value. As a result the Fab3H6 titers reach a plateau that can be achieved by improving either t_1/2 Deg _or q_Pi_. However the efforts by which the two parameters have to be varied are different, the degradation has to be changed by the factor of 4-6 and the intracellular protein formation by the factor 2.

## Discussion

Kinetic models describing the cellular fluxes of recombinant secreted proteins are available as well as their mathematic solutions [19,20]. However, in their practical application these models have usually been simplified by omission of parameters like dilution by growth as well as intracellular degradation. In our experiments we showed that q_Deg _and q_Dil _are of substantial dimensions, together being 65% of all intracellular synthesized protein. Therefore, especially if a model should be applied for further strain improvement applications, it appears to be essential that those parameters are considered in the model at least in microbial expression systems.

Growth conditions like specific growth rate, media composition or oxygen limitations, just to name some, affect recombinant protein production and thus alter its intracellular flux [27,28] So, it is necessary that the fluxes are measured under defined and reproducible conditions. In our case this is done in carbon limited chemostat cultivations using chemically defined minimal medium. Labeling with the non-radioactive and inorganic sulfur-isotope ^34^S, administered as sodium sulfate, enables the usage of bioreactors and a standard minimal production medium without amino acids. The common protein labeling procedures with radioactively labeled amino acids like ^35^S and ^3^H do not meet these demands.

Continuous labeling does not require any washing and centrifugation steps of the biomass and thus avoids the physiologically undefined conditions during this procedure. Non-labeled ^32^S and labeled ^34^S are measured simultaneously and thus the continuous labeling can be also interpreted as a chase of ^32^S. Therefore the same amount of information like with a pulse-chase experiment using common ^35^S amino acid labeling can be obtained. However, the detection limits, mainly depending on the resolution of the mass spectrometry, might be higher.

For efficient labeling, all sources of sulfur "contaminations" have to be avoided. This means that the sulfur in the media, and the trace salt solution within, has to be reduced to only one source, the labeled sodium sulfate. In our case the necessary changes were minimal, just the anions of the salts had to be adapted. The remaining sulfate concentration in the culture broth was quantified by nephelometric analysis with BaCl_2_. It contained about 10% of the sulfate of the feed medium. The amount of pulsed sodium sulfate has to be chosen due to labeling and osmolaric considerations. The non-labeled sulfate should be low and the change in osmolarity should not affect the protein fluxes. With the addition of 600 mg labeled sodium sulfate, the ^32^S concentration was below 1% throughout the experiment and the osmolarity increase was not detectable.

With this experimental setup the time constants and the corresponding half times of intracellular formation, intracellular degradation, dilution by growth as well as for secretion of the product can be determined. In the case of the recombinant secreted Fab3H6 35% of the protein is secreted wereas the majority (58%) is intracellularly degraded. The half time of protein degradation is 45.8 minutes, which is similar to the average half time of *S. cerevisiae *proteins, being 43 minutes [26]. The half time of secretion of 75.3 minutes seems to be rather slow, compared to e.g. carboxypeptidase in yeast [29]. However, the antibody fragment Fab3H6 is a heterodimer and thus could be more difficult to assembly and to secrete. In NS0 cells, Yee et al. [30] reported a half-time of one hour for IgG secretion for the major fraction of (70%) of IgG molecules. The remaining amount had a half-time close to the doubling time of NS0.

The major application of the model should be the support of a systematic strain improvement process. Therefore three dimensional sensitivity analyses were performed (figure 4). The impact of each parameter on overall expression titers as well as their synergistic effect was estimated. A ten fold improvement of the degradation or secretion half time resulted in two to three fold increased protein titers, respectively. Similar fold changes are often achieved when protein folding is engineered, like via *PDI1 *or BiP / *KAR2 *over-expression [14,15]. The protein synthesis rate, however, affects the secreted protein titer in a linear manner. By increasing the gene copy numbers, ten fold or higher protein production rates have already been achieved [3,31]. The linear correlation between gene copy numbers and expression levels may only be valid as long as no bottlenecks in folding or secretion occur [4]. Figure 4D takes a limitation in the secretory pathway into account. As a result protein titers reach a plateau, where further increase in the protein formation rate has no more effect. In such situations cell engineering of the secretory pathway may be the only way to break through the plateau by opening the secretion bottleneck.

Sensitivity analysis gives hints which parameter is most worthy to be modified in this regard. However strain development is an iterative process and it is likely that the change of one parameter also varies the others. Therefore after each engineering step the new fluxes should be determined. So we believe that the continuous ^34^S labeling described in this work may be a valuable tool for systematic strain improvement processes and deeper understanding of large scale production in bioreactors.

## Methods

### MATLAB implementation of the model and sensitivity analysis

The implementation of the model was done in MATLAB (additional file 1: MATLAB implementation of the kinetic model), and in its framework it is based on the work by Bibila et al. [22,32]. The model is formulated as set of ordinary differential equations, which are then solved over time using an ODE solver. For our secretion model we used an implicit linear multistep solver (MATLAB ode15s), because it is more appropriate for chemical or biochemical problems than an explicit Runge-Kutta pair solver [33].

To evaluate the behavior of the model concerning the input parameters, namely degradation, secretion and intracellular protein formation, always two of these parameters were varied against each other. The values were then plotted against the extracellular Fab3H6 concentrations in a three dimensional representation. The three dimensional plots were created using the MATLAB functions meshgrid and surf (additional file 2: Sensitivity analysis of the kinetic parameters).

### Yeast strain

The *P. pastoris *strain X-33 used in this study expressed the antibody fragment Fab3H6, previously described by Baumann et al. [27] and Dragosits et al. [34]. Both antibody chains are under the control of the constitutive GAP-promoter and are secreted via the *Saccharomyes cerevisiae *α-mating factor secretion signal. Fab3H6 is the anti-idiotypic antibody of the HIV neutralizing antibody 2F5. It has a molecular weight of 47.38 kDa and has 16 sulfur containing amino acids, 11 cysteines and 5 methionines [35,36].

### Chemostat cultivation

A preculture was incubated at 28°C for 24 h and 180 rpm on YPG (per liter: 10 g yeast extract, 20 g peptone, 10 g glycerol). The culture was harvested by centrifugation, resuspended in 50 ml sterile batch medium and used to inoculate 1.0 L batch medium in the bioreactor (Minifors, Infors, Switzerland) to a starting optical density (OD600) of 1.0.

After a batch phase of approximately 24 hours the continuous culture was started at a dilution rate of D = 0.1 h^-1 ^with a corresponding feed medium and harvest flow rate of 100 g h^-1^. Cultivation conditions were controlled constantly, the temperature at 25°C, pH at 5.0 with 25% ammonium hydroxide and pO_2 _at 20% by controlling the stirrer speed between 600 and 1200 rpm. Air flow was kept constant at 1.5 vvm (volume gas per volume medium and minute).

The batch medium contained per liter: 40 g glycerol, 2.0 g citric acid, 12.6 g (NH)_2_HPO_4_, 0.5 g MgSO_4 _• 7 H_2_O, 0.9 g KCl, 0.022 g CaCl_2 _• 2 H_2_O, 2 ml biotin stock solution (0.2 g L^-1^) and 4.6 ml PTM1 trace salt stock solution. The pH was set to 5.0 with 25% HCl. The PTM1 trace salt stock solution contained per liter: 65.0 g FeSO_4 _• 7 H_2_O, 20.0 g ZnCl_2_, 6.0 g CuSO_4 _• 5 H_2_O, 3.36 g MnSO_4 _• 1 H_2_O, 0.82 g CoCl_2 _• 6 H_2_O, 0.2 g Na_2_MoO_4 _• 2 H_2_O, 0.08 g NaI, 0.02 g H_3_BO_3 _and 5 ml H_2_SO_4 _(95 - 98%).

In the chemostat medium sodium sulfate was used as the only sulfur source. Per liter this medium contained 1.0 g citric acid monohydrate, 55 g glucose • 1 H_2_O, 9.83 g (NH_4_)_2_HPO_4_, 0.41 g MgCl_2 _• 6 H_2_O, 0.29 g Na_2_SO_4_, 1.7 g KCl, 0.01 g CaCl_2 _• 2 H_2_O, 2.0 ml biotin stock solution (0.2 g L^-1^) and 1.6 g PTM2 trace salt stock solution. The trace salt stock solution PTM2 contained (per liter): 63.3 g FeCl_2 _• 6 H_2_O, 20.0 g ZnCl_2_, 5.77 g CuCl_2 _2H_2_O, 3.94 g MnCl_2 _• 4 H_2_O, 0.82 g CoCl_2 _• 6 H_2_O, 0.2 g Na_2_MoO_4 _• 2 H_2_O, 0.08 g NaI, 0.02 g H_3_BO_3 _and 5 ml HCl (32%).

For the continuous labeling enriched ^34^S sodium sulfate (isotopic distribution: < 0.1% ^32^S, 1.1% ^33^S, 98.8% ^34^S and < 0.05% ^36^S) from Isoflex USA was used in the chemostat medium.

### ^34^S labeling

Cells were grown for at least 5 resident times in chemostat to ensure steady state conditions. Continuous ^34^S labeling was started by changing the feed to the ^34^S enriched medium. In addition, at the same time, a labeled sodium sulfate pulse, 5 mL sterile solution containing in total 600 mg of ^34^S-labeled sodium sulfate, was administered into the bioreactor. The change in the ^32^S to ^34^S ratio of the intra- and extracellular Fab3H6 was followed for 8 hours.

### Sampling

Samples were taken to determine the yeast cell dry mass, the extra- and intracellular Fab3H6 concentration and for immunoprecipitation of extra- and intracellular Fab3H6. During the ^34^S labeling samples were taken up to 6 times per hour. In this cases sample volume has to be kept small and therefore no biomass analysis was performed thereof.

The dead volume of the harvest port is 5 mL and therefore the first 5 mL culture broth were withdrawn. 10 mL culture broth were used for yeast dry mass (YDM) determination. For intracellular Fab3H6 measurements four cell pellets of 2 mL culture (0.05 g YDM each in capped screw tube, Biozym) were collected (1 min centrifugation at 4°C and 13.000 rpm, followed by quick freezing in liquid nitrogen). The supernatant was used for all extracellular measurements.

To enable the necessary sampling volume of at least 13 mL, the continuous harvest was replaced by discontinuous sampling. The sample volumes taken out from the bioreactor were exactly the volumes that should have been harvested by the pump. This caused a slight variation of the preset dilution rate of 0.1 h^-1^. However, the calculated dilution rates are in the range of minimum 0.0998 h^-1 ^and maximum 0.1013 h^-1 ^(calculation not shown) and it can be assumed that these changes have no significant influence.

### Mechanical cell lysis of *P. pastoris*

The cell pellets, containing 0.05 g YDM in capped screwing tubes, were washed with 0.5 mL PBS (per liter: 8.0 g NaCl, 0.2 g KCl, 1.8 g Na_2_HPO_4 _• 2 H_2_O, 0.24 g KH_2_PO_4_) and further resuspended in 0.5 mL lysis buffer. 0.5 mL of glass beads (acid washed, 0.4-0.6 mm, Satorius) were added. Cells were mechanically disrupted by using the FastPrep system (MP Biomedicals; settings: 3 times 20 s shaking at 6.5 m s^-1^). At the bottom of the tubes small holes were pierced with a hot needle and the tubes were put onto a 2 mL eppendorf tube. Cell lysates were collected by moderate centrifugation (1 min with 1.000 g). Cells debris and glass beads were washed with 0.5 mL lysis buffer followed by the same moderate centrifugation step. Lysates were cleared by centrifugation (15 min at 13.000 g) and the supernatants were taken for further analysis (Fab immunoprecipitation or quantification). During the whole procedure samples were kept at maximum 4°C.

Lysis buffer: 1% (w/v) triton X-100, 50 mM Tris·HCl pH = 7.4, 300 mM NaCl, 5 mM EDTA and 0.02% (w/v) sodium azide. Immediately before use inhibitors were added: 1 tablet of protease inhibitor (Sigma, S8820) per 20 mL buffer, proteasome inhibitor MG-132 to a final concentration of 5 μM and lysosomal inhibitor chloroquine to a final concentration of 50 μM.

### Fab3H6 immunoprecipitation

The procedure was adapted from the Current Protocols in Molecular Biology, chapter 10.16 "Immunoprecipitation" [37]. 80 μL of the anti-human IgG agarose suspension (Sigma-Aldrich) were added to 1 mL cleared cell lysate or 1 mL of culture supernatant and incubated for 2 hours at 4°C in a tube rotator. The agarose slurry was transferred onto PVDF membranes (Ultrafree^®^-MC Centrifugal Filter Units, 0.45 μm, Millipore) and washed four times with 0.5 mL ice-cold washing buffer and twice with 0.5 mL ice-cold PBS (centrifuged each time 5 sec at 3.000 g). To disaggregate the proteins from the matrix, the agarose suspension was incubated two times for 10 minutes with 120 μL elution buffer at room temperature. The eluates were collected by centrifugation (5 sec at 3.000 g). The buffer was changed to PBS and the solution was concentrated by the factor 10 by using the 10 kDa Amicon^® ^Ultra-0.5 centrifugal filter devices (Millipore). The immunopurified Fab3H6 solutions were used for sulfur isotope determination (see below).

Wash buffer: 0.1% (w/v) triton X-100, 50 mM Tris·HCl pH = 7.4, 300 mM NaCl, 5 mM EDTA and 0.02% (w/v) sodium azide. Immediately before use 0.1% sodium deoxycholate was added.

Elution buffer: 6 M guanidine hydrochloride, 100 mM Tris·HCl pH = 8.5, 5 mM EDTA and 0.02% (w/v) sodium azide.

### Sulfur isotope ratio determination

An inert titanium HPLC gradient system (Rheos 2000, Flux Instruments AG, Basel, Switzerland) with a metal-free autosampler (HTC PAL Autosampler, Thermo Fisher Scientific Inc., Waltham, USA) was used in combination with a high resolution inductively coupled plasma sector field mass spectrometer, ICP-SFMS (Element 2, Thermo Scientific Inc., Bremen, Germany). Sample introduction system consisted of a nebulizer (PFA-ST, Elemental Scientific Inc., Omaha, Nebrasca, USA) and a cooled (5°C) cyclonic silica glass spray chamber (PC3, ISA Elemental Scientific). Measured isotopes were ^34^S and ^32^S. Mass resolution was set to 4000. ^32^S was used as lock mass during measurement for instrumental mass drift correction. Dwell time per isotope was 0.1 sec. For separation by size exclusion chromatography a KW402.51E column (Shodex, Showa Denko K. K., Kawasaki, Japan) was used. Column dimensions were 1 × 150 mm. Separation was carried out under native conditions, with a 50 mM ammonium acetate, pH 6 eluent. The SEC flow was 50 μL min^-1^; injection volume was set to 2 μL. The integration of all chromatographic data from SEC-ICP-SFMS analysis was carried out using Chromeleon software (Version 6.7, Dionex, Sunnyvale, California, USA). Fab monomer and heterodimer dimer coeluted from the SEC column under the selected conditions. The ^34^S/^32^S ratio in the Fab samples was determined with a long term repeatability of 5% (12 hours, N = 5).

### Sulfur isotope determination of the inorganic compounds in the supernatant

The supernatant of the culture broth has been separated from all molecules with a size larger than 3 kDa by ultrafiltration (3 kDa Amicon^®^, Millipore). The sulfur in the remaining solution, mainly from inorganic sulfate, was analyzed by ICP-SFMS for its sulfur ratio (see above).

### Biomass determination by dry cell mass

Two times 5 mL culture broth were centrifuged. The pellets were resuspended in 10 mL RO-H_2_O (reverse osmosis water) and recentrifuged. The washed pellets were again resuspended in RO-H_2_O, transferred to weighed beakers and dried at 105°C until constant weight.

### SDS-PAGE and silver staining

10 μL of the IP concentrate were run on a non-reducing sodium dodecyl sulfate (SDS) 4 to 12% polyacrylamide gel (Invitrogen) with MOPS buffer (morpholinepropanesulfonic acid) at 200 V for one hour and silver stained according to the protocol in the Current Protocols in Molecular Biology, chapter 10.6 [38].

### Fab3H6 quantification

Quantification was done by sandwich ELISA as described in a previous study [27].

## Abbreviations

K_Deg_: time constant for intracellular protein degradation; K_Dil_: time constant for protein dilution; K_Har_: time constant for protein harvest; K_Sec_: time constant for protein secretion; P_e_: extracellular protein concentration; P_i_: intracellular protein concentration; P^32^S: concentration of the ^32^S isotope containing protein; q_Deg_: intracellular protein degradation rate; q_Dil_: protein dilution rate; q_Har_: protein harvest rate; q_Pi_: intracellular protein formation rate; q_Pi32S_: intracellular protein formation rate of the ^32^S isotope containing protein; q_Sec_: protein secretion rate; t_1/2 Deg_: half time of intracellular protein degradation; t_1/2 Dil_: half time of protein dilution; t_1/2 Sec_: half time of protein secretion.

## Competing interests

The authors declare that they have no competing interests.

## Authors' contributions

MP performed most of the experimental work, formulated and solved the kinetic model, and drafted the manuscript. MM contributed to study design, data analysis and interpretation. GK and SH designed and performed ICP-MS analysis of sulfur isotopes. ABG implemented the kinetic model in MATLAB and set up a MATLAB script for sensitivity analysis. DM conceived of the study and contributed to data analysis, interpretation and manuscript writing. All authors read and approved the final manuscript.

## Supplementary Material

Additional file 1 **MATLAB implementation of the kinetic model**. Time courses of the intracellular and extracellular labeled Fab concentrations after start of labeling are modeled dependent of the kinetic parameters of protein formation, degradation and secretion. Continuous cultivation (constant YDM and dilution rate) are prerequisits for numerically solving the differential equations. Click here for file

Additional file 2 **Sensitivity analysis of the kinetic parameters**. At a time two of the parameters, namely degradation, secretion and intracellular protein formation are varied against each other to determine the effect on extracellular Fab3H6 concentrations. Three dimensional plots are automatically created using the MATLAB functions meshgrid and surf. Click here for file
